# Criticality, Connectivity, and Neural Disorder: A Multifaceted Approach to Neural Computation

**DOI:** 10.3389/fncom.2021.611183

**Published:** 2021-02-10

**Authors:** Kristine Heiney, Ola Huse Ramstad, Vegard Fiskum, Nicholas Christiansen, Axel Sandvig, Stefano Nichele, Ioanna Sandvig

**Affiliations:** ^1^Department of Computer Science, Oslo Metropolitan University, Oslo, Norway; ^2^Department of Computer Science, Norwegian University of Science and Technology (NTNU), Trondheim, Norway; ^3^Department of Neuromedicine and Movement Science, Norwegian University of Science and Technology (NTNU), Trondheim, Norway; ^4^Department of Clinical Neuroscience, Umeå University Hospital, Umeå, Sweden; ^5^Department of Neurology, St. Olav's Hospital, Trondheim, Norway; ^6^Department of Holistic Systems, Simula Metropolitan, Oslo, Norway

**Keywords:** criticality, connectivity, neural disorder, *in vitro* neural networks, complexity, neuronal avalanches, neural computation, plasticity

## Abstract

It has been hypothesized that the brain optimizes its capacity for computation by self-organizing to a critical point. The dynamical state of criticality is achieved by striking a balance such that activity can effectively spread through the network without overwhelming it and is commonly identified in neuronal networks by observing the behavior of cascades of network activity termed “neuronal avalanches.” The dynamic activity that occurs in neuronal networks is closely intertwined with how the elements of the network are connected and how they influence each other's functional activity. In this review, we highlight how studying criticality with a broad perspective that integrates concepts from physics, experimental and theoretical neuroscience, and computer science can provide a greater understanding of the mechanisms that drive networks to criticality and how their disruption may manifest in different disorders. First, integrating graph theory into experimental studies on criticality, as is becoming more common in theoretical and modeling studies, would provide insight into the kinds of network structures that support criticality in networks of biological neurons. Furthermore, plasticity mechanisms play a crucial role in shaping these neural structures, both in terms of homeostatic maintenance and learning. Both network structures and plasticity have been studied fairly extensively in theoretical models, but much work remains to bridge the gap between theoretical and experimental findings. Finally, information theoretical approaches can tie in more concrete evidence of a network's computational capabilities. Approaching neural dynamics with all these facets in mind has the potential to provide a greater understanding of what goes wrong in neural disorders. Criticality analysis therefore holds potential to identify disruptions to healthy dynamics, granted that robust methods and approaches are considered.

## Introduction

Researchers have long grappled with the question of how the brain is able to process information, and many have recently turned to studying brain dynamics armed with tools from statistical physics and complexity science. In many physical systems, such as magnetic or gravitational systems, certain macroscopic features arise from the interactions of the constituent elements in a way that is unpredictable even from a perfect understanding of the behavior of each component; this is known as emergence (Chialvo, 2010). In the context of the brain, emergent phenomena encompass behavior and cognition, arising from the interaction of the vast number of neurons in the brain. Approaching the study of neural systems from this perspective entails studying neuronal behavior at the network or population level—observing and understanding emergent behaviors in the system rather than zeroing in on the behavior and connections of each individual neuron on its own. While exhibiting some computational power on their own, neurons are truly remarkable in their computational capacity when taken collectively.

It is hypothesized that the cortex may optimize its capacity for computation by self-organizing to a critical point (Beggs, 2008; Chialvo, 2010; Plenz, 2012; Shew and Plenz, 2013; Cocchi et al., 2017; Muñoz, 2018; Wilting and Priesemann, 2019a). Criticality is a dynamical state poised between order and disorder, or, more precisely, a transition between an absorbing phase in which activity gradually dies out and an active phase in which activity perpetuates indefinitely (Brochini et al., 2016). Critical systems must necessarily contain a large number of interacting non-linear components, though these conditions are not sufficient to ensure criticality; in the space of possible system states, criticality occupies a vanishingly small region, with chaotic and quiescent systems at the two opposite extremes (Brochini et al., 2016; Muñoz, 2018). A system operating in the critical state shows complex spatiotemporal behavior, and there is no scale, in space or time, that dominates the behavioral patterns of the system. That is, taking a closer or wider view of the system will show some variant of the same snapshot of the behavior. This mode of behavior is manifested by spatial and temporal correlations scaling as a power law over several orders of magnitude, giving rise to the presence of self-similar fractal-like structures over many scales. The brain exhibits complex spontaneous activity that crosses many time scales, a feature associated with criticality, and this activity is postulated to contribute to how the brain responds to stimuli and processes information.

In this review, we highlight network features evidenced to contribute to the emergence of critical dynamics in neural systems and discuss the benefits of experimentally studying the interplay between these features. Crucially, we also note here that care must be taken when extrapolating from theoretical findings on criticality to the more recent experimental research on criticality in neural systems observed at different scales. In particular, experimental explorations of criticality in neural systems point to the importance of considering the structures (Massobrio et al., 2015) and plastic mechanisms (Ma et al., 2019) that support this dynamical regime. Thus, critical dynamics in neuronal networks may be better understood by characterizing their connectivity and how this connectivity changes over time or in response to inputs and perturbations. Additionally, as discussed in detail by Shew and Plenz (2013), there is also much to be learned about the computational and functional benefits that criticality confers; thus, complementing graph theoretical and criticality metrics with an information theoretical approach can further shed light on the functional benefits of this dynamical regime.

Note that we aim here to focus on relevant considerations for empirical assessments of criticality in biological neural systems, particularly at the network level, and on how experimentalists may build upon the existing theoretical foundations to address the criticality hypothesis from a data-driven perspective. This review thus aims to provide the reader with basic insights on criticality and how it relates to neuroscience, rather than an in-depth discussion of the physics of criticality. Furthermore, we approach modeling studies with an eye on how they can inform our understanding of experimental systems but do not exhaustively review the vast field of model neural systems, as this is a topic of review unto itself.

In the remainder of this section, we present an overview of the theoretical benefits of criticality and experimental evidence supporting its emergence in living neural systems. The next section then focuses on the intersection between network neuroscience and criticality and discusses the connectivity features that can support critical dynamics. In the subsequent section, we consider the plasticity mechanisms that allow these networks to form, learn in response to inputs, and remain stable against perturbation or failures in the network. Finally, we conclude with a discussion of how approaching the study of criticality with a diversity of perspectives may prove more fruitful than any single directed approach.

### Why Is Criticality Important?

The term self-organized criticality was coined as such to reflect the similarity of this phenomenon with the critical point observed in phase transitions in statistical mechanics, wherein a parameter, such as temperature, can be tuned to bring the system to a state between multiple phases of matter (Bak et al., 1988). However, a crucial point that distinguishes self-organized criticality from the conventional critical point in statistical mechanics is that the system tunes *itself* to criticality without the need for external tuning via a control parameter. In a self-organized critical system, the critical point is an attractor, meaning the system tends to evolve toward that point from a wide range of starting points; to again consider the parallel with thermodynamic criticality, if a thermodynamic system were to show self-organized criticality, intrinsic mechanisms would drive the system to return to the critical point between the liquid, solid, and gaseous phases. Obviously this is not the case, as matter in each of these phases can exist stably, but there are many fascinating properties conferred by criticality, as we will discuss in this section.

Many natural systems have been observed to show critical or critical-like behavior (Paczuski et al., 1996; Chialvo, 2010), including forest fires (Malamud et al., 1998; Buendía et al., 2020; Palmieri and Jensen, 2020) and flocks of birds (Cavagna et al., 2010), which has led researchers to explore the possibility of a similar phenomenon in the brain in a conjecture known as the criticality hypothesis (Beggs and Plenz, 2003; Beggs, 2008). This hypothesis states that the brain self-organizes into the critical state in order to optimize its computational capabilities.

The canonical sandpile model by Bak et al. (1987, 1988) describes a system slowly driven by the addition of grains of sand until an instability occurs and the sand is redistributed to restabilize the system. Because of the dynamical minimal stability of the system, the chain reactions set off by the external drive, with sand traveling from site to site until the system restabilizes, called “avalanches,” display the self-similar power-law scaling mentioned above. This means that the disruption of a single element in the system has a small but non-zero chance to change the state of the whole system. Work on emergent properties of dynamical systems has indicated that systems tuned to the critical regime show optimal information processing capabilities (Langton, 1990; Shew et al., 2009, 2011; Plenz, 2012; Shew and Plenz, 2013).

To provide a conceptual image of the complex behavior that can arise from even very simple parts and lay a foundation for how computation can emerge in such a system, we consider as an illustrative example the binary cellular automaton (CA), a system consisting of many stationary binary cells whose behavior is influenced by the states of the cells in their immediate neighborhood. Box 1 gives a brief definition of the CA and summarizes the important findings obtained by Langton (1990) in his work on how computation may emerge in physical systems at the “edge of chaos.” It should be noted, however, that although this regime was initially assumed to exhibit a continuous second-order phase transition, as classically described for critical dynamics, it has recently been found to show a discontinuous first-order transition (Reia and Kinouchi, 2014, 2015). In a vanishingly small region in the state of all possible CAs of the type Langton (1990) considered, quite interesting behavior emerges: complex patterns of activity are preserved over long distances in space and time. In the regime between the two extremes of quiescence and disorder (Kinouchi and Copelli, 2006), the system optimizes its capacity to perform the functions of information transmission, modification, and storage that are necessary to support computation.

Box 1 **Cellular automata at the “edge of chaos.”**

A binary cellular automaton (CA) is an *n*-dimensional array of binary cells whose states are updated synchronously in discrete time steps. The state of each cell at time *t* + 1 depends on the states of the cells in its neighborhood at time *t*. Such CAs are among the simplest systems to show complex behavior. Langton (1990) used the binary CA as a lens to assess the conditions under which a physical system may show the capacity to support computation. In a sweep of the possible rulesets for a one-dimensional binary CA, he demonstrated that a small subset of rules produce behavior compatible with the necessary tenets of computation, namely, the storage, transmission, and modification of information.

**Figure 1:**
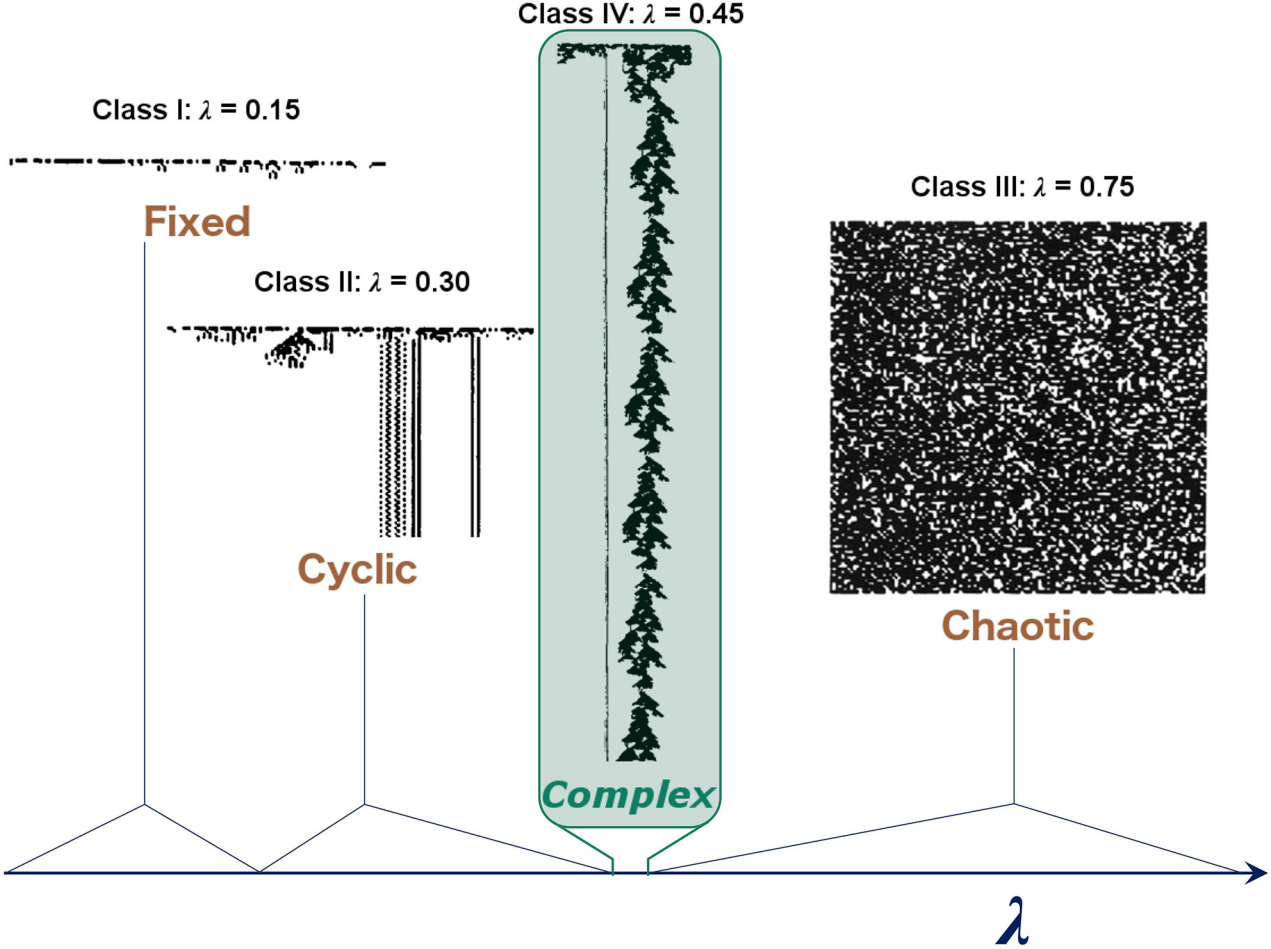
Illustrative examples of the behavior observed in different classes of one-dimensional binary CAs. In these CAs, each row represents the CA at a given time step, and the two states of the cells are represented by black and white. Complex behavior arises in the critical regime, which becomes vanishingly small as the system increases in size. Langton (1990) characterized these CAs with the λ parameter, which represents the ratio of transitions to an arbitrary state selected as the “quiescent state.” Adapted from Langton (1990).

Examples of different “classes” of CA (Wolfram, 1984) corresponding to different dynamical regimes are shown in Figure 1, with class IV representing a transitional state analogous to criticality. He also demonstrated that these CAs occupy a small region, where mutual information is maximized at a point of intermediate entropy. This maximal mutual information indicates that these CAs at the “edge of chaos” have struck a balance between the competing needs of information storage, which requires low entropy, and information transmission, which requires high entropy, thereby allowing complex patterns of activity to propagate through the system over time and space without rapidly dying out or overwhelming the system. Despite its simplicity, the CA demonstrates how some sets of rules balancing quiescence and transmission can lead to complex patterns that allow for the transfer of information, an appealing property for neural systems.

Although the transition hypothesized to occur in neural systems is distinct from the “edge-of-chaos” transition shown in Box 1, the features captured in this simpler system can help us understand why the dynamics at phase transitions may be relevant for information processing in the brain. In the transitional regime, patterns of activity are preserved over space and time, which means that spatially disparate elements of the system can communicate with each other and that informational representations are propagated in time. Different inputs produce distinguishable outputs, allowing systems near criticality to respond to stimuli in a meaningful way. These concepts underlie how information is encoded and transmitted in dynamical systems at criticality and highlight how studying criticality in experimental studies on neural computation can inform our understanding of how the brain processes information.

What does this have to do with computation? In a general sense, computation is a process, either natural or artificial, by which information is communicated and manipulated to produce some kind of meaningful behavior in a system (Denning, 2007). More concretely, computation is the act of solving a “computational problem”: a set of related questions with given information (input), each with its own distinct answer (output). Criticality has been found to optimize characteristics related to better performance at solving computational problems. For example, recurrent network models showing critical dynamics outperform their sub- and supercritical counterparts in terms of their input-to-output mappings; that is, the outputs produced from different inputs are more separable, or distinguishable, in critical networks (Bertschinger and Natschläger, 2004). Critical systems also show a maximal dynamic range (Kinouchi and Copelli, 2006; Gautam et al., 2015), which is the span of inputs distinguishable by the system. Additionally, the number of metastable states is maximized in networks with a critical branching ratio (Haldeman and Beggs, 2005), where a metastable state is defined as a cluster of similar output patterns produced by the same input. Information transfer and storage, represented, respectively, by the information shared between a source node and a destination node and that between a node's past and future states, is also optimized at criticality (Boedecker et al., 2012).

Although criticality is recognized to optimize many properties associated with computation, as discussed above, it should also be noted here that there are also some properties associated with criticality that may run counter to computational function in the sense described here (Wilting and Priesemann, 2019a). For example, the maximal dynamic range of a system in the critical state causes the specificity of the system to suffer; that is, a system that can sensitively respond to a wide range of inputs also shows more overlap between responses to similar inputs (Gollo, 2017). Thus, recent research has shifted from a singular focus on criticality to a broader realm of dynamical possibilities, including heterogenous networks composed of both critical and slightly subcritical subgroups (Gollo, 2017), the presence of a “reverberating regime,” enabling the task-dependent switching or combining of critical and slightly subcritical dynamics to enjoy the benefits of both states (Wilting et al., 2018), and the concept of self-organized quasi-criticality, which accounts for non-conservative dynamics in systems that show critical-like behavior over a finite range of scales (Bonachela and Muñoz, 2009; Bonachela et al., 2010; Buendía et al., 2020; Kinouchi et al., 2020). These findings show promise for the advancement of a more detailed and physically accurate view of how criticality is realized in living neural systems; however, we refrain in this review from venturing too far into the details of these topics and direct the interested reader to the cited literature.

### Experimental Evidence of Criticality in Neural Systems

**Figure 2:**
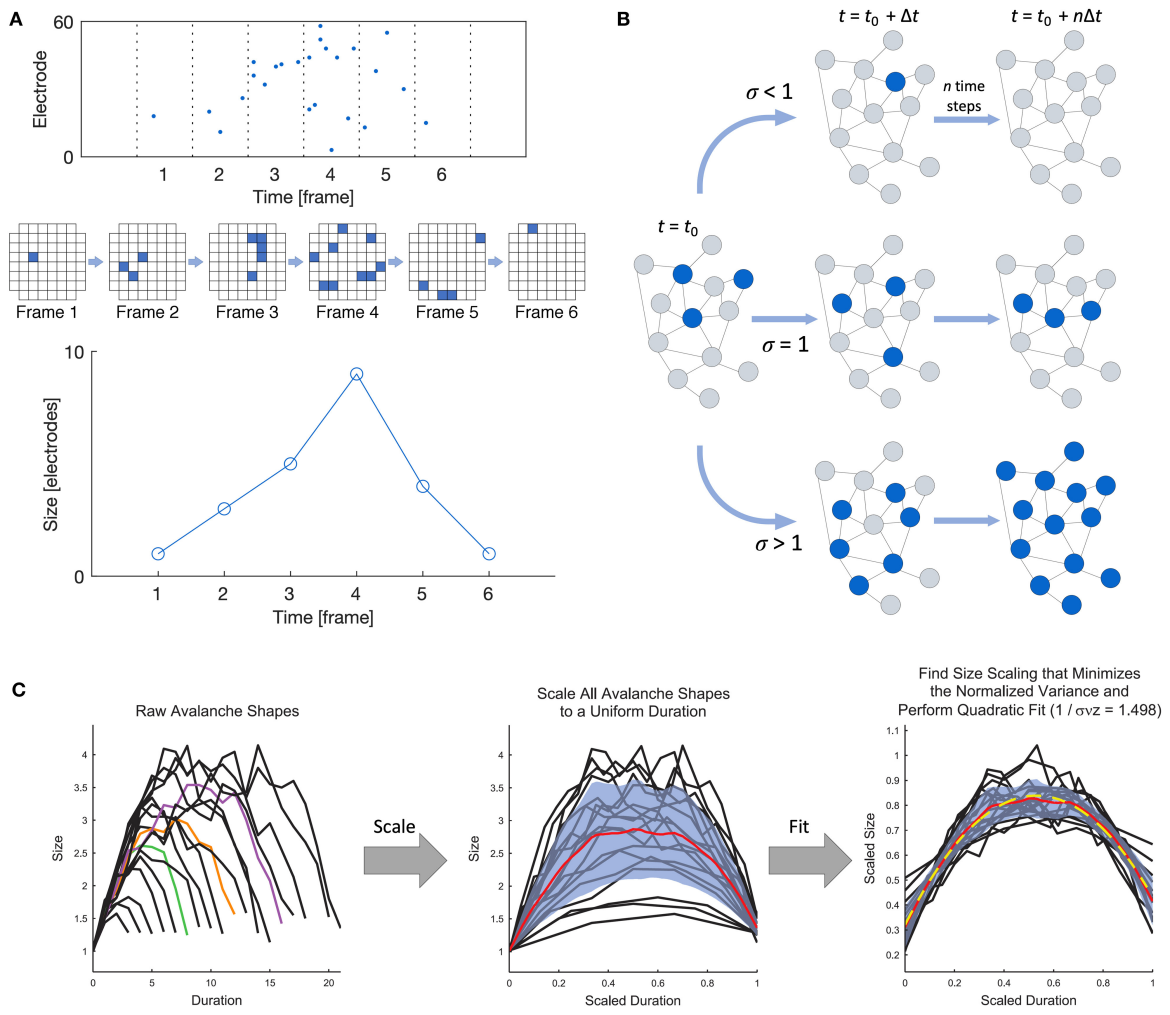
Definition of a neuronal avalanche and examples of empirical measures of criticality. **(A)** Definition of a neuronal avalanche. The top panel shows a raster plot divided into time bins, and the avalanche in the plot spans six active frames preceded and followed by inactive frames. An alternate view of the activity in the six frames is shown below, where each square represents an active electrode in an 8 × 8 grid. The bottom panel shows the definition of the avalanche shape, which is obtained by taking the number of active electrodes in each frame. **(B)** Illustration of the branching ratio. Blue nodes are active, and gray are inactive. A branching ratio of 1 allows activity to persist without overwhelming the system. **(C)** Shape collapse. In a critical system, all avalanches should show the same mean temporal shape profile across different size scales. Adapted from Marshall et al. (2016).

Beggs and Plenz (2003) were the first to experimentally demonstrate that the spontaneous behavior of *in vitro* cortical networks displays features consistent with critical dynamics in their landmark study on neuronal avalanches in cortical slices interfaced with microelectrode arrays (MEAs). At its most general, a neuronal avalanche extends over the duration of persistent activity propagating through the network and is punctuated by silent periods preceding and following the active period, as shown in Figure 2A. In the case of *in vitro* systems (i.e., slices or dissociated cultures), “activity” may refer to either the higher-frequency spikes or the lower-frequency local field potentials (LFPs), as both modalities have been studied (e.g., Beggs and Plenz, 2003; Pasquale et al., 2008). Criticality has also been studied at the macroscale using electroencephalography (EEG) (e.g., Meisel et al., 2013; Lee et al., 2019).

Regardless of the scale or method of data collection, one main hallmark of criticality is that neuronal avalanches show power-law scaling in both space and time, with sub- and supercritical behavior being characterized by exponential and bimodal distributions, respectively. Beggs and Plenz (2004) demonstrated that neuronal avalanches show diverse spatiotemporal patterns that are stable over several hours, highlighting their capacity to represent a wide range of information in a reproducible manner. It should be noted that a simple power-law fitting alone is not sufficient to identify criticality (Goldstein et al., 2004; Priesemann and Shriki, 2018); in fact, this was far from the only approach used by Beggs and Plenz (2003), who also evaluated the branching ratio and the effect of using only a subset of all recording points. In addition to the methods first used by Beggs and Plenz (2003), a number of criticality measures have since been put forward. Providing empirical evidence of criticality is challenging, and it is suggested that a range of measures be applied (Priesemann and Shriki, 2018); we present a selection of some such measures in Box 2.

Box 2 **Experimental metrics of criticality.**

To pursue further investigation of the criticality hypothesis, we must be armed with the appropriate tools for identifying a network's dynamic state. Power laws are notoriously challenging to handle in empirical data (Clauset et al., 2009) and can also arise in non-critical systems (Martinello et al., 2017; Touboul and Destexhe, 2017; Priesemann and Shriki, 2018). Thus, additional measures are needed to accurately identify when a network is in the critical state, and each measure should also be applied to appropriate null models for comparison. This box lists the main approaches currently used to identify criticality from empirical data, but it should be noted that the development of such methods remains an active area of research. Some of the measures listed below have been implemented in a freely available MATLAB toolbox called the Neural Complexity and Criticality Toolbox (Marshall et al., 2016), and detailed statistical analysis for fitting and analyzing power-law distributions can be performed with an open Python package called powerlaw (Alstott et al., 2014).

- *Power-law scaling of neuronal avalanches*: One hallmark of criticality in neuronal networks is the power-law scaling of the size *S* and duration *T* of neuronal avalanches. That is, *P*(*S*) ∝ *S*^−α^ and *P*(*T*) ∝ *T*^−β^, where *P*(·) is the probability distribution function. The size is generally defined as the number of activated electrodes or neurons, and the duration is the number of active time bins. When the time bin width is selected to correspond to the average inter-spike interval, the power law exponents of the size and duration have been shown to be approximately α = 1.5 and β = 2.0. However, the power-law scaling should persist across a range of temporal resolutions close to the order of magnitude of the average inter-spike interval, with the exponent α changing systematically with the selected time bin size (Beggs and Plenz, 2003; Pasquale et al., 2008). Power-law scaling should also remain when a more coarse-grained spatial resolution is considered, by using only a subset of all recording points. As stated above, there is an open Python package called powerlaw that can be used for detailed statistical analysis of power-law distributions (Alstott et al., 2014). As an additional power-law related metric, the κ parameter (Shew et al., 2009) gives a quantitative measure of the difference between the experimental and fitted cumulative probability distributions when using power-law fitting.
- *Branching ratio*: The branching ratio σ is the ratio of the number of descendants to the number of ancestors, where activity on an ancestor electrode or neuron immediately precedes activity on a descendant electrode or neuron (Beggs and Plenz, 2003). A system in the critical state has a branching ratio of approximately 1, allowing activity to flow through the network without dying out (σ <1) or overwhelming the entire network (σ > 1), as shown in Figure 2B. A modified version of the branching ratio that is specific to LFP data has also been introduced, where the ratio is instead taken between the baseline-to-baseline areas of the negative LFP deflections (nLFPs) in successive time bins, rather than the number of nLFPs (Plenz, 2012). The nLFP area is correlated with the number of neurons firing and thus provides a better measure of group activity during an avalanche than the nLFP count.
- *Shape collapse*: When a system is in the critical state, avalanches should show the same mean temporal profile across scales. The temporal profile of an avalanche represents the number of active sites as a function of time, and for a system in the critical state, the temporal profiles of all avalanches collapse onto the same profile shape when spatiotemporally scaled with a scaling exponent γ close to 2 (Figure 2C), as described by 〈*S*〉(*T*) ∝ *T*^−γ^, where 〈*S*〉(*T*) is the average size of all avalanches of a given duration *T*. Details can be found in Sethna et al. (2001) and Friedman et al. (2012), and an experimental demonstration of shape collapse in non-human primates can be found in Miller et al. (2019). The deviation from criticality coefficient (DCC) by Ma et al. (2019) is related to the concept of shape collapse and is computed from the difference between the scaling exponent γ calculated from empirical data using linear regression and the expected value calculated from the power-law exponent α of the size distribution.
- *Spatial subsampling*: Because of the nature of observing neuronal systems, only a subset of the system components can be sampled. This spatial subsampling can sometimes lead to erroneous conclusions about the nature of the system's underlying dynamics. Methods involving the scaling of spatial subsampling (Levina and Priesemann, 2017) and a subsampling-invariant estimator (Wilting and Priesemann, 2018) have been developed to allow for the evaluation of dynamic states of subsampled systems.
- *Other measures*: Some researchers have developed other quantitative measures to describe the dynamical state of the system. One notable example is the use of statistical scaling laws related to a phenomenon called “critical slowing down,” which refers to the tendency for systems to require more time to recover from a perturbation the closer they are to criticality (Meisel et al., 2015a). Additionally, detrended fluctuation analysis (DFA) offers a framework to understand scale-free oscillations in a range of systems (Hardstone et al., 2012).

Avalanche behavior during development was first observed in organotypic cortical cultures by Stewart and Plenz (2008), and they found that avalanches persisted throughout development over periods of up to 6 weeks *in vitro*, despite large changes in activity levels, suggesting homeostatic regulation to maintain this mode of activity. It has also been demonstrated that dissociated cortical networks may self-organize into the critical state after a period of maturation, though not all such networks reach the critical state and reports on the time course of maturation differ (Pasquale et al., 2008; Tetzlaff et al., 2010; Yada et al., 2017). The reported results on dissociated networks suggest that after a period of low activity, networks tend to pass through periods of first subcritical then supercritical behavior before settling into the critical state, though not all networks reach this state. This behavior has been hypothesized to stem from an initial overproduction of connections followed by a period of pruning excess connections (Pasquale et al., 2008; Yada et al., 2017). Additionally, experiments in which chemical perturbation is applied to increase excitation or inhibition in the network indicate that networks at criticality exhibit a balanced excitation-to-inhibition (E/I) ratio (Shew et al., 2009, 2011; Heiney et al., 2019). Together, these experimental findings point to the importance of a balance in both network structure and network dynamics to achieve criticality.

Shew et al. (2009, 2011) have explicitly linked the dynamic state of a cortical network with its information processing capacity by demonstrating that networks at criticality show maximal dynamic range, information transmission, and information capacity in comparison with their counterparts in the sub- and supercritical states. These properties harken back to the original requirements posed by Langton (1990) for a system to be capable of computation and further emphasize the role of the dynamical state in governing the functional behavior of a neuronal network. These studies highlight the functional benefits conferred by the critical state and give credence to the criticality hypothesis (Shew and Plenz, 2013). But how does a system organize itself to become capable of supporting critical dynamics? In the following sections we explore the relationship between the structure of a network and its dynamical behavior and consider the plasticity mechanisms that form and maintain target structures.

## Criticality and Networks

Biological neural networks are interconnected networks of individual information processing units (neurons). When considering how information is processed within the network, it is vital to understand the interactions of the individual units, the organization of the brain network, and the integration of activity of widely distributed neurons (Bressler and Menon, 2010; van den Heuvel and Sporns, 2013). Underlying the aggregate activity of groups of neurons are the structural and functional connectivity of the network, which determine where signals pass and which neurons act in consort (Sporns, 2002; Womelsdorf et al., 2007). This in turn influences the information processing capabilities of neural networks, and network structure therefore contributes to determining the emergence of critical properties in neural networks. The question is then how the organization of biological neural networks can support critical dynamics to optimize computational efficiency. This section examines a selection of experimental and simulation-based studies that address this question.

### Network Neuroscience

The application of modern network science to the brain and networks of neurons has flourished over the past two decades. The complex network of the brain has information processing as its primary goal and attempts to maximize this capacity while under multiple constraining influences, such as availability of space, energy, and nutrients, and thus must strike a balance between computational capacity and wiring cost (Laughlin and Sejnowski, 2003; Cuntz et al., 2010). These two factors are often in a tradeoff relationship; for example, direct connections facilitate the most effective signal transmission, but the long-range connections this requires are very costly to grow and maintain (Buzsáki et al., 2004). Additionally, the network must meet the changing demands of the organism while remaining resilient to damage to or failure of parts of the network, such as the loss of neurons or the connections between them (Pan and Sinha, 2007). Some of the basic network science principles that are commonly applied in neuroscience studies are highlighted in Box 3.

Box 3 **Benefits of network topology.**

Network neuroscience encompasses an approach to studying brain function that considers the ways in which neurons communicate, anatomically and functionally, across multiple scales (Bassett and Sporns, 2017). It is informed by complex systems theory, which states that the emergent behavior of a system cannot necessarily be understood simply by the properties of its individual components. It further applies mathematical techniques such as graph theory and algebraic topology to describe networks (graphs) in terms of their individual units (nodes) and their connections (edges). A node in this context can be a brain area, a single neuron, a recording electrode, a voxel, pixel, or any unit which describes the activity of a discrete part of a neural network. Edges can be physical connections obtained from connectivity mapping or functional connectivity based on correlation or other measures (Bullmore and Sporns, 2009). This approach has yielded great insight into how the brain is organized and how communication within brain networks occurs (Newman, 2003). While numerous methods exist for extracting the structural, functional, effective, weighted, or binary networks from living neural systems (for a review see Bullmore and Sporns, 2009; Bastos and Schoffelen, 2016; Hallquist and Hillary, 2018), there are several features among these considered to play an important role in neuronal organization and function.

**Figure 3:**
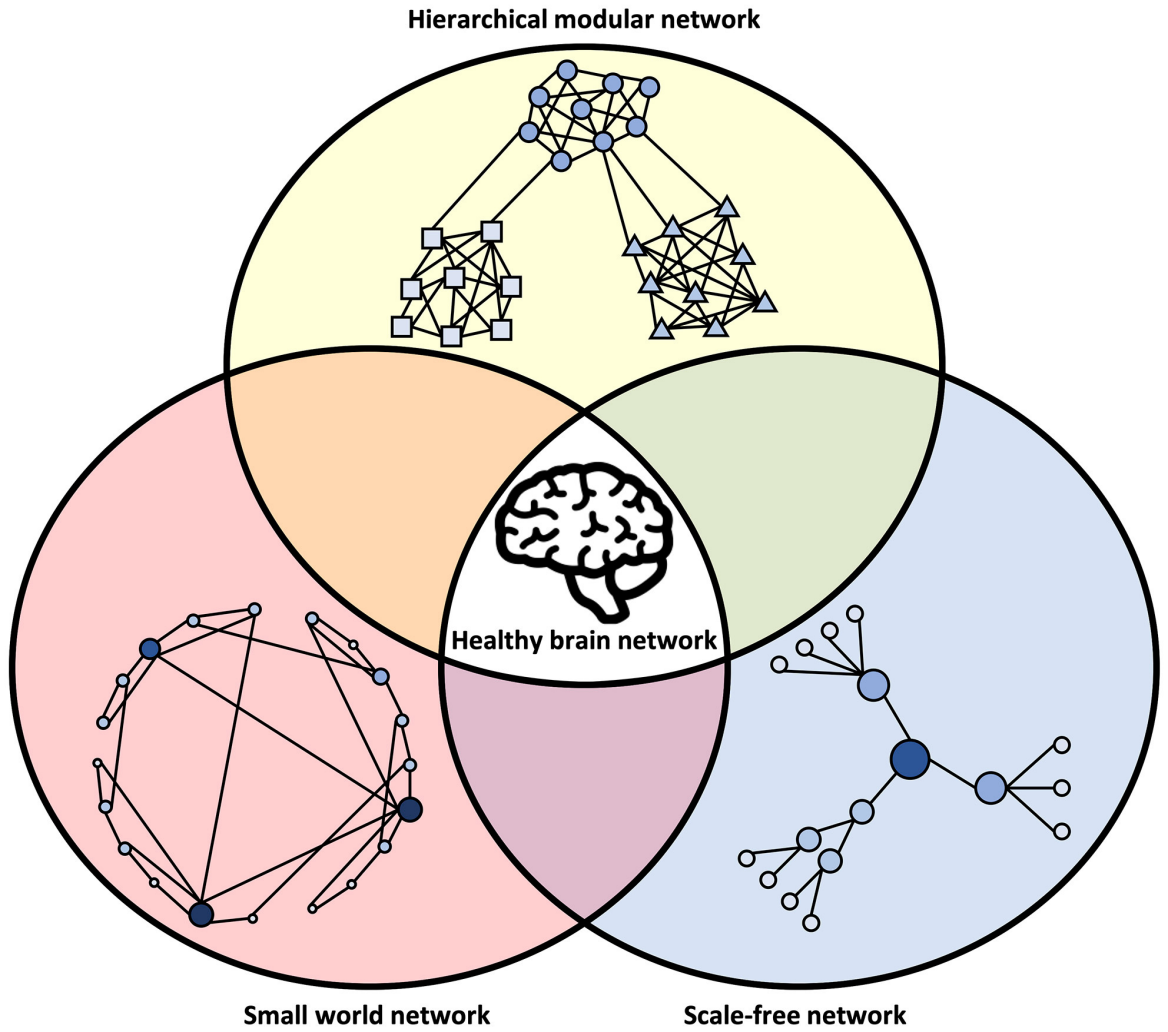
Network characteristics associated with healthy brain networks. In normal conditions, brain networks show hallmarks of multiple network models. This includes an intermediate state between order and randomness in small-world organization, the power-law degree distribution of a scale-free network, and modular clusters organized in a hierarchical fashion. The integration of these different network types may be an evolutionary adaptation driven by the multi-constraint optimization of brain wiring. Deviations from the hallmarks of these network structures may be associated with abnormal brain function and disease.

*Small-World Network*: A small-world network is typically defined by how closely it approaches the small world ideal of high clustering and low characteristic path length (Watts and Strogatz, 1998). One way to produce a small-world network is to begin with a regular (or lattice) network, where each node is connected only with its nearest neighbors, and, with probability *p*, rewire each connection in the network to a randomly chosen node elsewhere in the network. When *p* = 1, every connection is rewired, and the result is a random network. However, at intermediate rewiring probabilities, the characteristic path length drops off drastically, showing that only a few long-range connections are necessary to facilitate the integration of the network. Additionally, the clustering of nodes remains high, retaining the local specialization of the original regular network. These properties make small-world networks highly advantageous for computation while reducing wiring cost (Chklovskii et al., 2002), essentially reducing the number of connections without sacrificing the capacity for network-wide communication (Bassett and Bullmore, 2017).
*Scale-Free Network*: In a scale-free network, the probability distribution of the node degree, which is the number of edges connected to each node in the network, follows a power law, meaning most nodes have a small number of edges and few nodes have many (Barabási and Albert, 1999; Eguíluz et al., 2005). These high-degree nodes are often called hubs, and they serve an important role in integration across the network (Sporns et al., 2004). Hubs make a scale-free network more robust to random deletion of nodes but susceptible to targeted damage of the hub nodes (Albert et al., 2000). Especially vulnerable (but not exclusive to scale-free networks) is the rich club (Zhou and Mondragón, 2004), a group of hubs with a high degree of interconnectivity between each other. While there is growing evidence for the presence of rich-club topology in the brain (Griffa and Van den Heuvel, 2018; Kim and Min, 2020) the presence of scale-free topology (Bonifazi et al., 2009) is still somewhat controversial, but it provides important insight in modeling studies of dynamics on network topology (Broido and Clauset, 2019).
*Hierarchical Modularity*: A modular network is characterized by the presence of clusters of nodes that are densely connected with each other and share few edges with nodes outside the cluster. In a hierarchically modular network, these clusters can be subdivided into other clusters according to the same principle, often over multiple scales (Figure 3). Modules are interconnected by connector nodes, which may or may not be hub nodes, allowing dissemination of signals and integration of information across the system. Modular networks may be more robust to dynamic change within the network. The intricacies of hierarchical modularity and its relation to other network topologies such as the rich club (McAuley et al., 2007) are extensive and, as such, beyond our scope here (for a review, see Meunier et al., 2010).
  It is important to consider that the network models described above are not mutually exclusive. Rather, it appears that the brain displays hallmarks of all these network types (Bullmore and Sporns, 2009) and that deviations from their properties can be involved in disease (Stam, 2014), as illustrated in Figure 3.

Multiple lines of evidence now show that brain networks have a small-world organization, with high local clustering and low average path length, which facilitates segregated local specialization and global integration. Low-cost, short-range connections dominate, while a smaller number of long-range connections allow for few intermediaries between distant components (Bullmore and Sporns, 2012). Brain networks also show evidence of link clustering, where strong connections preferentially form between nodes with similar neighborhoods (Pajevic and Plenz, 2012). Some evidence also indicates brain networks are scale-free, with a heavy-tail degree distribution that follows a power law (Eguíluz et al., 2005). Components with high degrees furthermore tend to connect to other high-degree components in a “rich club,” which are hub regions of high connectivity that facilitate integration across distinct areas and wide propagation of signals and information (Sporns, 2013). The central nervous system (CNS) is also divided into specialized areas at multiple levels, from brain lobes to smaller but separate modules within these lobes, which can again be subdivided into further modules. This is characteristic of hierarchical modularity, which facilitates flexibility in adaptation because it can incorporate changes within a single module without affecting other, nearby modules. This makes the system at the same time robust and flexible (Meunier et al., 2010). The combination of these network architectures—small-world, scale-free, and modular—creates an efficient network well-suited for computation, as will be discussed in the following section.

### Neural Network Topology Facilitates Criticality

Modeling work has provided evidence that the network features outlined above contribute to the emergence of critical dynamics as a means to support computation in networks of neurons. This provides some motivation to translate these findings into the experimental realm, but little work has been done thus far in this regard, despite the expanding experimental work on criticality, as already detailed above, and the large body of work on network neuroscience (Bassett and Sporns, 2017). However, one noteworthy methodological study has identified small-world organization in the effective (causal) connectivity of a cortical slice culture (Pajevic and Plenz, 2009). With further applications of such measures of connectivity to assess avalanche propagation *in vitro*, it will be possible to evaluate if network features found to be beneficial in modeling studies, such as small-worldness, can be experimentally confirmed. In this section, we will examine criticality and complex network features, but it should also be noted that criticality can also be demonstrated in random (Kinouchi and Copelli, 2006; Costa et al., 2015; Campos et al., 2017) and complete (Levina et al., 2007; Bonachela et al., 2010; Brochini et al., 2016; Costa et al., 2017; Kinouchi et al., 2019; Girardi-Schappo et al., 2020) networks. In a study specifically examining the impact of network structure on network dynamics *in silico*, Massobrio et al. (2015) showed that random network topology can only support power-law avalanche scaling under a narrow range of synaptic constraints and firing rates. Furthermore, of the topologies they investigated, only scale-free networks with a high average node degree and small-world features were able to display behavior consistent with experimental criticality.

Simulation studies on complex networks have found that features of criticality can emerge with biologically plausible regulatory mechanisms. Shin and Kim (2006) found that, for a network initialized as complete, i.e., fully connected, and allowed to change its connections over time by spike-timing-dependent plasticity (STDP), the network reorganizes into a scale-free network with small-world properties that shows evidence of self-organized criticality. Other studies have also found that concurrently scale-free and small-world networks recapture critical dynamics with exponents comparable to those found experimentally (Lin and Chen, 2005; Pellegrini et al., 2007; de Arcangelis and Herrmann, 2012). Complementary to this, Rubinov et al. (2011) demonstrated that a hierarchically modular structure with a preponderance of within-module connections, which have a relatively low wiring cost, produced a much broader critical regime than was observed in corresponding non-hierarchical networks. It has also been shown that in the Bak–Tang–Wiesenfield (BTW) model, also referred to as the “sandpile” model, self-organized criticality emerges as a result of the formation of modular clusters with biologically relevant dimensions, lending further evidence to the importance of modular network structures (Hoffmann, 2018).

When focusing on the activity of ensembles of neurons, it is common to consider bursting activity that encompasses multiple units and how these units coordinate their activity. In dissociated cortical neurons, networks that spontaneously develop critical dynamics display a level of synchrony higher than what is seen in uncoordinated subcritical activity but lower than that seen in highly regular supercritical activity (Pasquale et al., 2008; Valverde et al., 2015; Cocchi et al., 2017). This is also consistently reported in modeling studies and can be related to the branching ratio, or how many downstream neuronal responses are elicited by a single active neuron (Box 2). When the branching ratio is balanced near 1, the network is in a state of intermediate synchrony and tends to display critical avalanche dynamics in a way that maximizes the number of adaptive responses the network can produce to stimulus (Haldeman and Beggs, 2005; Shew and Plenz, 2013). However, despite bursting activity often being considered highly coordinated, critical networks *in vitro* have been observed to show more burst-dominated activity than their supercritical counterparts, with critical networks showing a higher proportion of spikes contained in bursts and an intermediate level of synchrony within those bursts (Pasquale et al., 2008).

A biological constraint for the branching parameter is the level of inhibition present as mediated by inhibitory interneurons (Girardi-Schappo et al., 2020). In the human cortex, 15–30% of neurons provide local inhibition, and this E/I ratio is frequently replicated in *in silico* models by tuning the number of inhibitory nodes and their connectivity within the network (Rudy et al., 2011; Tremblay et al., 2016). In both models and biological networks, inhibitory nodes typically constrain their connectivity within modules or clusters. In examinations of this inhibitory connectivity, it has been found that local inhibitors are necessary for critical dynamics in systems combining modularity and plasticity (Rubinov et al., 2011). Furthermore, Massobrio et al. (2015) tested a wide range of E/I ratios on scale-free networks and were only able to achieve critical dynamics in networks with inhibitory nodes comprising 20–30% of all nodes. They also observed the effect of the E/I ratio of the hub nodes specifically and found that the same ratio of approximately 30% inhibition in the hubs was able to support critical dynamics across a wide range of mean degrees, whereas none of the fully excitatory hub networks displayed critical behavior. In models and in the brain, this balance of excitation and inhibition acts as a countermeasure against runaway excitation and stabilizes the network dynamics (Fingelkurts et al., 2004; Shin and Kim, 2006; Meisel and Gross, 2009; Naudé et al., 2013; Salkoff et al., 2015). Furthermore, through the careful tuning of the E/I balance, multiple dynamic states can also be achieved in the same model (Li and Shew, 2020).

### The Brain May Operate in a Critical Region, Not at a Critical Point

While the criticality hypothesis of the brain is attractive because it provides a model for brain activity that optimizes information processing and storage, aspects of the model are difficult to reconcile with knowledge of the brain's activity. For instance, the brain's activity and dynamics are not constant but fluctuate widely depending on multiple factors. That this widely variable and adaptable dynamic system can be tuned to a specific critical *point* can therefore seem counterintuitive. However, a finite system at criticality does not have to be tuned to a specific point but rather exhibits critical behavior over a particular region. The phase transition can be continuous, such that there exists a range of states within the system that support critical dynamics (Hesse and Gross, 2014). This extended model of criticality appears much more compatible with our knowledge of the brain's dynamics than the notion of a strict critical point. One such form of critical range is referred to as the Griffith's phase and appears to be facilitated by hierarchical, modular network architectures, which are consistent with the previously investigated small-world architecture of the brain (Gallos et al., 2012; Moretti and Muñoz, 2013; Ódor et al., 2015; Girardi-Schappo et al., 2016). A wide critical range would appear to be advantageous for the network, making the critical dynamics more robust against failure or perturbation than in the case where criticality can only be achieved in a narrow range or single point (Li and Small, 2012; Wang and Zhou, 2012). This range appears to be dependent on the level of structural heterogeneity or disorder within the network, including variance in the node in-degree distribution (Muñoz et al., 2010; Wu et al., 2019).

Additionally, as mentioned in the section on the importance of criticality, recent evidence suggests that a strict adherence to criticality may not be the sole aim of network organization (Wilting and Priesemann, 2019a). On the basis of these findings, it has been hypothesized that some brain networks may self-organize to points in a slightly subcritical range, where they could then flexibly tune their dynamics in accordance with the demands of a given task (Wilting and Priesemann, 2018; Wilting et al., 2018). Following this hypothesis, certain tasks may benefit from a reduced dynamic range in the network to subsequently reduce interference from non-task-specific inputs. Networks may also show heterogeneous local dynamical states, with a mixture of critical and subcritical regions balancing the competing demands for specificity and sensitivity (Gollo, 2017).

Although we have highlighted in this review how the underlying network structure may influence the emergence of critical dynamics here, it is not evident that these topological features are in and of themselves necessary for a network to be considered critical. As mentioned, simulation studies have found that power-law avalanche scaling can be obtained in regular, random, and small-world networks (de Arcangelis and Herrmann, 2012; Michiels Van Kessenich et al., 2016), and regardless of the edge directedness or the presence of inhibitory edges (Ódor and Kelling, 2019). Furthermore, it is possible to achieve power-law avalanche scaling in networks with only weak pairwise correlations and not the more complex patterns of functional connectivity seen in biological networks (Thivierge, 2014). The relationship between critical dynamics in the brain and its underlying network structure may therefore reflect a balance between computational capacity, the metabolic cost of the network activity (Thivierge, 2014), wiring cost in network development (Laughlin and Sejnowski, 2003; Cuntz et al., 2010), and the resilience of the network against perturbations (Goodarzinick et al., 2018). Though certain complex network topologies may better accommodate and broaden the range of critical dynamics (Li and Small, 2012; Moretti and Muñoz, 2013), they are only one component of a neural system.

## Plasticity is Necessary to Achieve and Maintain Criticality

**Figure 4:**
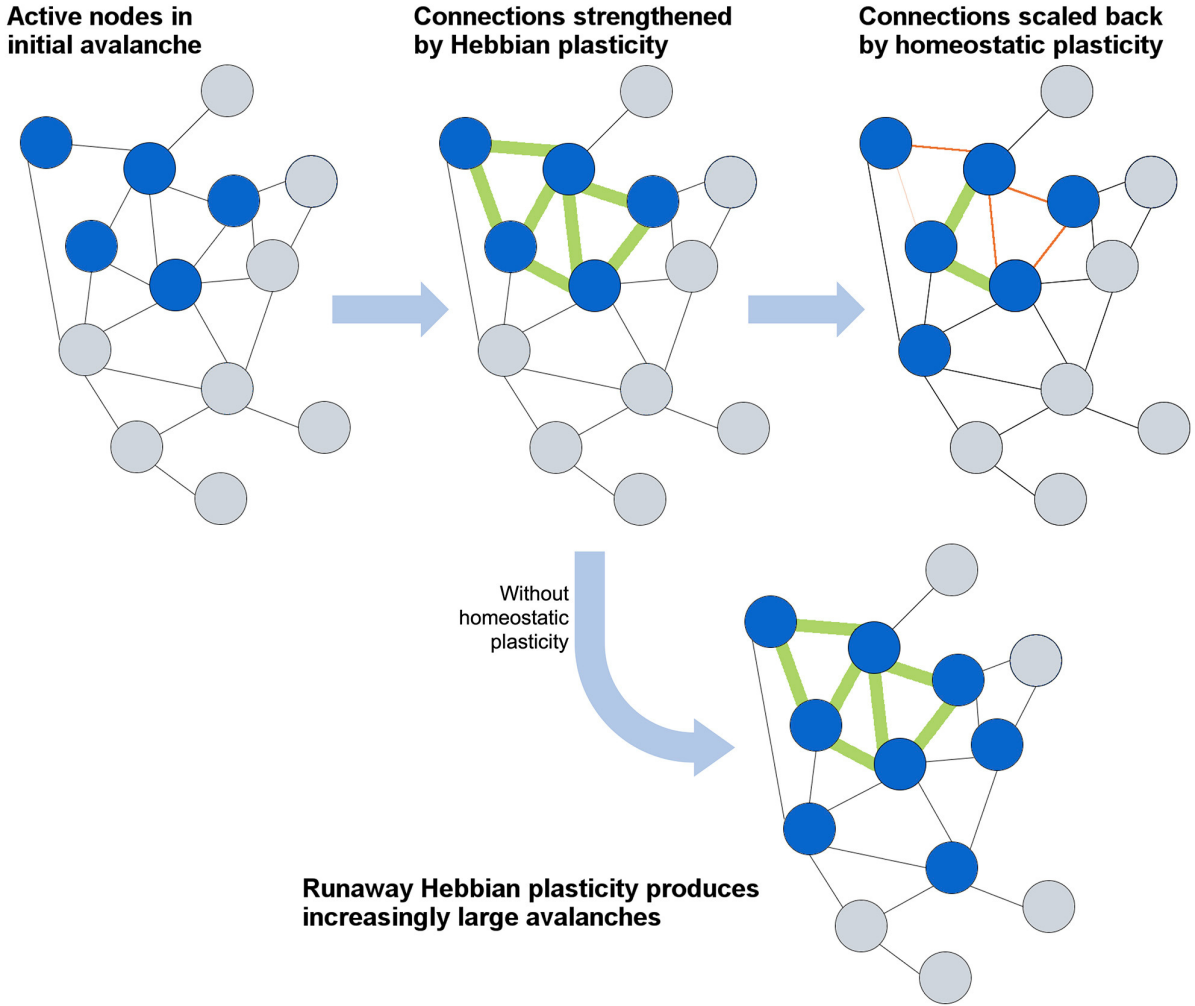
Schematic overview of the interplay between Hebbian and homeostatic plasticity. Hebbian plasticity serves to strengthen and form connections between neurons that fire together, whereas homeostatic plasticity maintains a balance in connections and activity levels. The lowermost case demonstrates how an absence of homeostatic plasticity would allow runaway Hebbian plasticity to overwhelm the network with activity.

How efficient network structures are formed in different dynamical systems varies widely from system to system, and numerous models have been developed to describe the growth of efficient networks. The first general model for scale-free network formation was proposed by de Solla Price (1965) and popularized by Barabási and Albert (1999). Through the addition of nodes as the network evolves, each new node is preferentially attached to an existing node with high connectivity, resulting in a “rich-get-richer” hub formation and power-law-distributed connectivity. However, such models cannot represent neural growth, as they forego an important consideration of neural network formation: self-organization into an efficient topology depends not only on the connectivity but also on synaptic strength, E/I ratio, and, vitally, the plasticity that defines all of these network parameters. In this section, we will first focus on the role of plasticity in activity-dependent network formation and then consider how networks maintain the critical state through homeostatic plasticity (Stepp et al., 2015). The interplay between activity-dependent and homeostatic plasticity is schematically illustrated in Figure 4.

### Establishing Critical Dynamics in Neuronal Networks

Whereas network models may be constructed in a variety of ways to display critical dynamics and scale-free structures, actual neuronal networks form and maintain connections under numerous constraints. It is generally acknowledged that during development, neurons overshoot the number of necessary connections and then go through a phase of pruning before reaching a relatively stable state of connectivity (Low and Cheng, 2006). There is also evidence that cortical networks *in vitro* go through this same sequence of overshoot and pruning as they mature, and after this stage they may exhibit critical dynamics, though not all networks do (Stewart and Plenz, 2006; Pasquale et al., 2008; Yada et al., 2017). Van Ooyen et al. (1995) and Okujeni and Egert (2019) showed that a simple axon growth model assuming activity-dependent radial growth could form a network similar to those found *in vitro* by utilizing activity spontaneously arising in the network. Even with only a simple activity-dependent growth rule applied to systems with random initial placement, these systems have been shown to grow into a state supporting avalanches with power-law scaling in the behavior of the final networks (Abbott and Rohrkemper, 2007; Kossio et al., 2018). Correspondingly, the trajectory of the dynamic state *in vitro* appears to move from a subcritical to a supercritical state before ultimately reaching criticality. As the supercritical state in this case produces network-wide synchrony, it is probable that plasticity mechanisms reduce the global excitation level as a result of this synchrony and drive the network toward criticality. To mimic some of this development, numerous models have attempted to generate critical networks using plasticity rules applied to random, small-world, and scale-free topologies (de Arcangelis et al., 2006; Rubinov et al., 2011; de Arcangelis and Herrmann, 2012; Teixeira and Shanahan, 2014; Michiels van Kessenich et al., 2018, 2019). These models typically apply local Hebbian mechanisms, such as STDP, to rewire the network into a weight distribution or topology capable of achieving critical dynamics, thus recapitulating certain facets of biological network development.

Simple plasticity mechanisms based on correlated firing, such as STDP, can shift the topology of networks by changing the connection weights, resulting in directed and more complex networks. In observations of activity-dependent neural development in computational models, a number of researchers have observed the same general trend: that these mechanisms tend to drive the dynamics toward criticality, with the resulting topologies showing scale-free organization (Bornholdt and Röhl, 2003; Meisel and Gross, 2009). The end result is robust against different initial topologies and changes to the underlying parameters, such as average connectivity. Even when initializing a network from a random topology, STDP is sufficient in some models to drive the network toward critical dynamics (Teixeira and Shanahan, 2014; Li et al., 2017; Khoshkhou and Montakhab, 2019). Li et al. (2017) have also investigated the computational benefit of these STDP-trained networks, which showed improved input-to-output transformation performance at criticality (see also Bertschinger and Natschläger, 2004; Siri et al., 2007, 2008 for the computational benefits of criticality and Hebbian plasticity in recurrent neural networks).

Recent studies have furthered this modeling approach with the addition of more neurobiologically relevant features, such as axonal delay and hierarchical modularity. The inclusion of a time or axonal delay between pre- and post-target activation can shift both the directionality and distribution of synaptic strengths from a bimodal to a unimodal distribution without a loss of critical dynamics (Khoshkhou and Montakhab, 2019). Note that the potential of Hebbian mechanisms to produce critical dynamics is still model-dependent and can also drive the network to supercritical states. Again, the complexity of the network topology plays a vital role in conjunction with these mechanisms, as modular and hierarchical topologies both can counteract this supercritical organization and broaden the critical regime once it arises (Rubinov et al., 2011). Though neural development is an immensely complicated and complex process, Hebbian mechanisms appear to be one of many aspects that play a vital role in the self-organization of neural networks toward criticality and supporting topologies.

### Homeostatic Maintenance of the Critical State

Although learning and developmental mechanisms, such as STDP, drive networks toward certain configurations, the network patterns formed in this way are not simply static structures but also undergo plastic changes to maintain homeostasis and in response to external stimuli. A number of researchers have investigated how different forms of plasticity influence the dynamical state of neuronal networks (Rubinov et al., 2011; Stepp et al., 2015; Zierenberg et al., 2018; Ma et al., 2019).

A network's resilience to damage necessitates a level of adaptability to restore dynamics following a perturbation beyond that offered by topologies that are robust against component failure (see Box 3). This adaptability is hypothesized to stem from homeostatic plasticity, which provides feedback to restore the overall excitability in local connections and the network. Whereas Hebbian plasticity is evidenced to give rise to critical dynamics, homeostatic plasticity is evidenced to maintain the network activity within this dynamic regime despite varying input levels and intrinsic activity (Levina et al., 2007; Naudé et al., 2013; Ma et al., 2019). This adaptive excitability can be exemplified through the branching ratio. A steadily increasing input level would produce branching parameters exceeding 1, given the increasing level of extrinsic excitation on the system. Yet by homeostatic scaling of the excitability in the network and the input connections, the branching parameter can be maintained, avoiding supercritical dynamics. The inverse also holds true in the absence of inputs. Homeostatic plasticity acts toward an intrinsic set point for the network's excitability and adjusts synaptic response to maintain input specificity (Turrigiano, 2017). In the absence of homeostatic plasticity mechanisms, such as synaptic scaling, Hebbian mechanisms create feedback loops of excitatory response that remove any specificity to synaptic input (Wu et al., 2020).

Michiels van Kessenich et al. (2018, 2019) have included global homeostatic plasticity mechanisms in their network models to observe if the networks are able to exhibit avalanches with power-law scaling and evaluate the performance of the network on classification tasks. By feeding error back into the network based on the desired outputs at certain readout sites, they were able to train the network to recognize different input patterns, including classifying handwritten digits. The response of the network to inputs after this training period showed a clear spatial organization, with distinct regions responding to different inputs. Although the network studied here is a simplified computational model, the results indicate that plasticity mechanisms are able to drive networks toward criticality and play a role in their capacity for learning and computation. Also, as shown by Girardi-Schappo et al. (2020), the use of *multiple* homeostatic mechanisms can generate highly diverse firing patterns and promote the self-organization of a network toward a critical point. Additionally, as with Hebbian plasticity and criticality, the application of homeostatic mechanisms can enhance the computational capabilities of the network by tuning it toward criticality, and increasing both the number of input patterns that can be distinguished by the network and the separability of these patterns (Naudé et al., 2013).

The role of homeostatic mechanisms in maintaining critical dynamics is exemplified experimentally in a study by Shew et al. (2015). There is growing evidence that homeostatic mechanisms such as synaptic depression aid in allowing the visual cortex to adapt to changes in sensory input and recover critical dynamics. Using *ex vivo* preparations of the visual cortex, Shew et al. (2015) demonstrated adaptation of the cortex to stimuli; upon first exposure to a stimulus, the network transiently showed non-critical dynamics, followed by a return to criticality via homeostatic plasticity. In a critically tuned model of their network, an external input similarly drove the network out of a critical state, and critical dynamics were then restored through the implementation of a synaptic scaling rule, indicating a likely mechanism for the homeostatic adaptation. While this provides evidence for short-term tuning toward criticality, there is also recent evidence of long-term homeostatic adaptation. Thus far, studies including homeostatic mechanisms in criticality experiments and models have largely been focused on such synaptic mechanisms or the E/I balance, with only scant focus on intrinsic plasticity (Naudé et al., 2013; Li X. et al., 2018; Zhang et al., 2019; Girardi-Schappo et al., 2020) or metaplasticity (Kinouchi et al., 2020, in preprint; Peng and Beggs, 2013).

The effect of E/I imbalance has been well-described in previous studies, as mentioned previously; however, manipulation of the intrinsic mechanisms underlying the E/I balance have largely been unexplored (Plenz, 2012). Ma et al. (2019) attempted to bridge this experimental gap by examining a well-established model of homeostatic plasticity in the context of criticality. By systematically exploring the space of possible E/I configurations, Ma et al. (2019) demonstrated that the balance achieved by critical networks may be struck with a number of configurations—specifically, they varied the E/I ratio, the number of excitatory neurons receiving input from each inhibitory neuron, and the ratio of inhibitory neurons receiving input. This shows that different possibilities exist for how networks may be configured to achieve criticality, though only a small fraction of the potential combinations yielded critical activity. Furthermore, when a combination of E/I parameters adjacent to one of the critical regimes was selected and further explored by allowing the network to evolve under synaptic scaling and STDP, it was unable to achieve critical dynamics regardless of the plasticity parameters, demonstrating the importance of local inhibitory dynamics in achieving criticality.

Crucially, when synaptic scaling was removed from the model by Ma et al. (2019), the model was no longer able to recover critical dynamics after a reduction in input. With synaptic scaling removed from the excitatory population, reduction in input resulted in runaway activity and increased synaptic strength due to uncompensated STDP; removing synaptic scaling from inhibitory neurons also shifted the network out of the critical regime, though less dramatically, and was accompanied by a reduction in synaptic strength. In contrast, removal of STDP left the network unaffected by the reduction in input, indicating the necessity of this type of plasticity for the network to give a meaningful response to inputs.

## Brain Disorders and Disruptions to Criticality

Up to this point we have examined criticality's relationship to the network topology that supports it and the plastic mechanisms that organize and maintain it. Because of the intricate interplay of these underlying mechanisms, disruptions to either topology or plasticity can manifest as deviations in the dynamic state of the network, and as such, criticality analysis may aid in the identification of such disruptions and provide a better understanding of the mechanisms at play. In this section, we will detail studies that apply criticality analysis to the identification and prediction of diseases and disorders in the nervous system and propose suggestions for how to expand this work moving forward. Here we use the term “perturbation” in a medical sense to refer to disruptive and negative impacts to a network's baseline state, such as the severing of axons *in vitro* or epileptic states *in vivo*.

### Criticality on Disrupted Foundations

**Figure 5:**
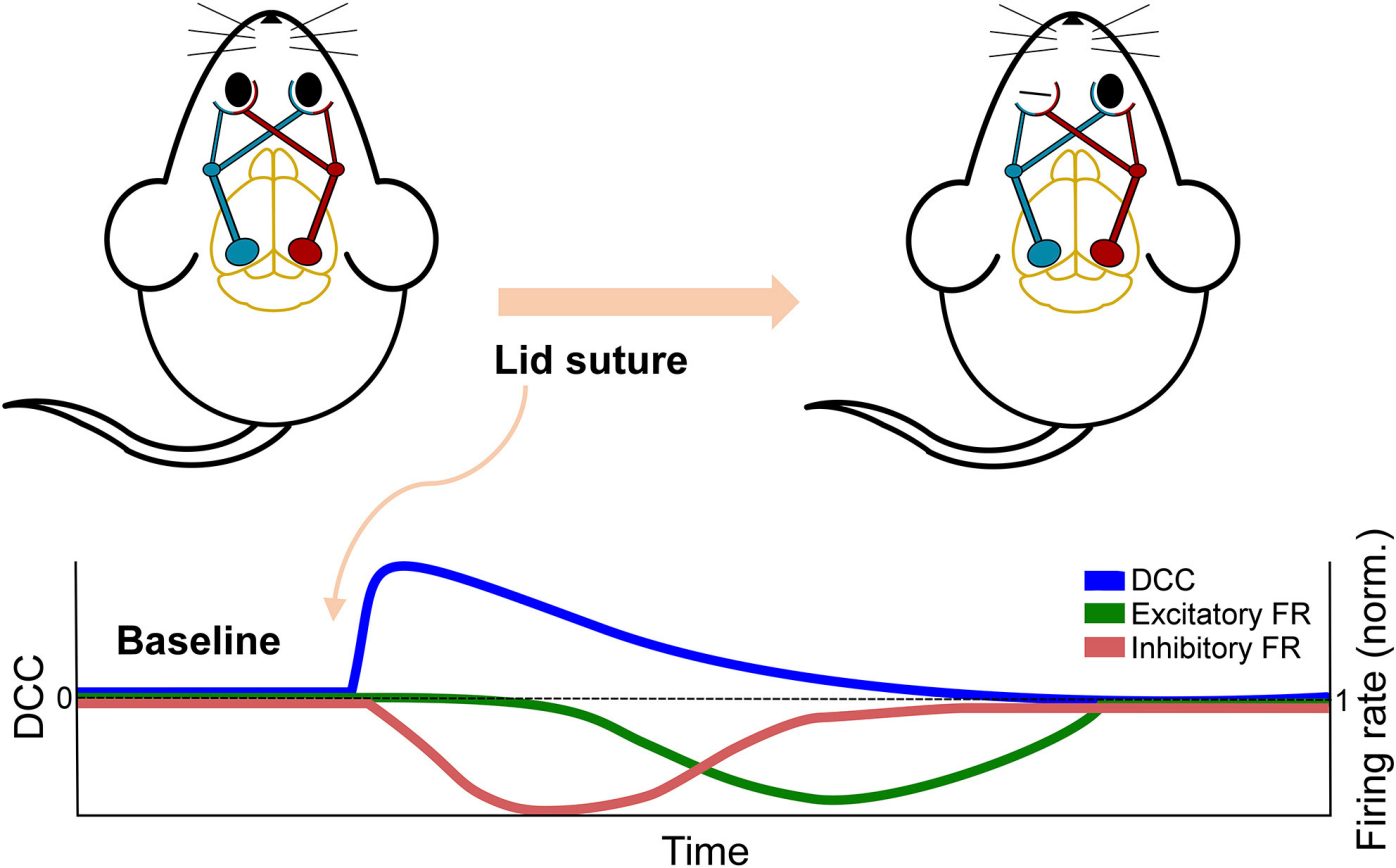
Schematic overview of *in vivo* experiment from Ma et al. (2019). The overall firing rate of the network showed a delayed response to the perturbation of removing excitatory sensory input by monocular deprivation. In contrast, the DCC and other criticality-related measures showed an immediate response and a more rapid return to baseline.

The study by Ma et al. (2019) discussed above in the context of plasticity also provides substantial experimental insight into the effect of perturbations on the dynamic state of neuronal networks. By inducing monocular deprivation in rodents with chronic recording of the visual cortex, they were able to examine the effect of the perturbation on cortical activity. Despite the near complete removal of input to the cortex, the network's firing rate, or activity level, was initially maintained, providing no evidence of the sensory deprivation that had occurred. However, the neuronal avalanche behavior in the network revealed a deviation from criticality immediately following perturbation, despite the fact that the firing rate was maintained. Moreover, this deviation was sustained until homeostatic mechanisms restored it by upscaling inhibitory activity and subsequently reducing network firing (see Figure 5). The deviation from criticality and branching ratio measures (see Box 2) applied by Ma et al. (2019) exemplify the capacity for criticality analysis to identify perturbations. Additionally, this study emphasizes how multiple mechanistic underpinnings lend themselves to critical dynamics and the potential disparity between network dynamics and global activity levels. The effect of these mechanisms can be further emphasized through their disruptions during a critical state.

Modeling studies on disrupted network topology and impaired plasticity lend some insight into deviations from criticality following perturbation. Within scale-free and small-world networks, there exists a significant robustness against structural defects, as most nodes only connect to neighbors within a cluster or module. As a result, a large number of these low-degree nodes can be lesioned before critical dynamics are disrupted (Goodarzinick et al., 2018). Conversely, any removal of high-degree nodes or long-range connections can rapidly fragment the network structure and subsequently abolish any critical dynamics occurring (Callaway et al., 2000; Mizutaka and Yakubo, 2013; Valverde et al., 2015). The network-wide synchrony that occurs with efficient network topologies and at criticality may also enable the spread of disruptive states such as epilepsy. This synchrony is also dependent on a functioning and adaptive E/I balance, as discussed below.

In investigations of criticality in biological neural networks, it has been found that the simple addition of the GABA inhibitor bicuculline can shift the dynamics of a network from critical to supercritical by increasing synchrony within the network (Beggs and Plenz, 2003; Pasquale et al., 2008). Other studies have also shown that altering the balance of excitation and inhibition in biological networks can drive them into a different dynamic state. In self-organized critical networks, pharmacologically enhancing excitation can change the dynamics of the network from critical to supercritical, while reducing excitation promotes subcritical dynamics (Shew et al., 2009, 2011). Furthermore, it has been demonstrated that direct inhibitory action by addition of GABA to a network with supercritical dynamics can drive it into a critical state (Heiney et al., 2019). This points to GABAergic inhibition as important in disrupting highly synchronized activity where activity very frequently propagates throughout the entire network, driving it into a supercritical state. Multiple simulation studies have also shown that emergence of critical dynamics is dependent on a certain proportion of inhibition in the network, which conform to physiological levels of inhibitory neurons in the brain (de Arcangelis et al., 2006; Massobrio et al., 2015).

### The Promise of Criticality Analysis in the Clinical Realm

Despite the rising interest in biological criticality in the last two decades, there has been a dearth of experimental and clinical studies connecting criticality to perturbations. Because criticality represents an optimal state for computation, one could expect departure from the critical state would entail a disruption unto itself (Shew et al., 2011); however, in practice, disruptions to network dynamics are likely more complicated than a transition away from criticality, as such transitions may be part of healthy activity (Stewart and Plenz, 2006; Pearlmutter and Houghton, 2009; Allegrini et al., 2015; Wilting and Priesemann, 2019b), though see also (Carvalho et al., 2020, in preprint) for a counterpoint to this. Given recent hypotheses concerning network computation in the slightly subcritical regime, quantifying the impact of perturbations on network dynamics necessitates rigorous analytical tools (see Box 2) (Priesemann et al., 2014; Wilting et al., 2018; Wilting and Priesemann, 2019a). The potential presence of heterogeneous local dynamics or global reverberating dynamics in the subcritical regime demands a combined and comparative approach for any medical application.

The existing literature detailing disorders as disrupted criticality largely pertain to the macroscale (see Zimmern, 2020 for a comprehensive review), as examined through (i)EEG (Thatcher et al., 2009), ECoG (Chaudhuri et al., 2018), and fMRI (Tagliazucchi et al., 2012). Even with the growing number of researchers examining the dynamics of mesoscale networks, there is still a lack of research turning these *in vitro* and *in vivo* methods toward the dynamics of perturbed networks. Given the growing sophistication of molecular and electrophysiological tools, the potential for experimental manipulation of network topology and homeostatic mechanisms is immense. In the absence of many mesoscale investigations into criticality perturbations (Stewart and Plenz, 2006; Gireesh and Plenz, 2008; Fekete et al., 2018), this section will focus on macroscale network dynamics and investigations into clinical disorders through the lens of criticality.

The majority of today's macroscale studies on disorders and criticality pertain to epilepsy disorder, which provides an example of how criticality analysis can benefit the clinical field (Worrell et al., 2002; Li et al., 2005; Meisel et al., 2015b, 2016; Arviv et al., 2016; Meisel and Loddenkemper, 2019; Rings et al., 2019). Given the absence of literature on other disorders in this context and the substantial literature that exists on network topology (Terry et al., 2012; Lopes et al., 2020) and E/I balance (Wei et al., 2017; Du et al., 2019) as they relate to epilepsy, we have chosen to examine this disorder here. An epileptic state, or seizure, is characterized by a departure from healthy dynamics to a hyper-synchronized or chaotic state. This epileptic state can be either focal and confined to cortical regions or circuits, or generalized and encompass the entire brain (Terry et al., 2012; Englot et al., 2016). Currently, diagnosing the presence of epilepsy is often based on the presence of overt structural deficits through MRI and CT, or through markers of infection and electrolyte testing (Stafstrom and Carmant, 2015). When the epilepsy is rooted in less overt factors, diagnosis becomes an issue of determining disruptive dynamics which has thus far proved to be a substantial problem (Stafstrom and Carmant, 2015; Meisel and Loddenkemper, 2019). To this end, the application of criticality analysis to epilepsy has been the focus of much recent research which we will discuss here (Meisel et al., 2015b, 2016; Arviv et al., 2016; Meisel, 2016; Beenhakker, 2019; Du et al., 2019; Meisel and Loddenkemper, 2019; Witton et al., 2019; Maturana et al., 2020).

Current literature links epileptogenesis to disruptions in network connectivity or neuronal excitability stemming from genetic pathologies, such as ion channel mutations, or acquired conditions, such as stroke (Terry et al., 2012; Wei et al., 2017). Predicting if and how these disruptions will lead to epileptogenesis has resulted in the development of measures of network excitability and synchrony and most recently the application of criticality analysis (Meisel, 2016). Diagnostically, criticality analysis has been applied as a biomarker in focal epilepsy patients, where critical slowing down, which is the stretching of activity patterns near a critical state, has been found to precede seizure onset (Maturana et al., 2020). Similarly, the presence of a Hopf bifurcation has been indicated as a diagnostic predictor based on modeled ECoG, neural field, and neural mass dynamics (Meisel and Kuehn, 2012; Buchin et al., 2018; Deeba et al., 2018); for a review on this topic, see Meisel and Loddenkemper (2019). In terms of treatment, the branching ratio computed from recordings of resting dynamics provides a quantitative measure to characterize the effect of anti-epileptic drugs (AEDs), as an alternative to the typically used absence of seizure or response to transcranial stimulation (Meisel et al., 2015b, 2016). A further study by Meisel (2020) also applied this to show an inverse correlation between network synchrony and AED dosage levels, indicating a shift toward a subcritical and away from a supercritical seizure state. However, we should note here that these promising results in the field also highlight some of the intricacies around criticality analysis. For example, when investigating critical slowing down and using a similar iEEG dataset as Maturana et al. (2020), Wilkat et al. (2019) conversely found no evidence of critical slowing down as an epileptic biomarker.

While these studies focus largely on neuronal excitability in isolation, the use of such analysis should also be integrated with the substantial work underway on epilepsy networks (Terry et al., 2012). Already, mapping of functional and structural connectivity in epileptic networks can identify the ictal, or seizure, onset zone and examine the spread from local to global seizure by means of effective connectivity (Yaffe et al., 2015). Indeed, models of seizure propagation and clinically recorded networks show rapid spread of disruptions through small-world networks as a result of their long-range connectivity and hub structure (Netoff et al., 2004; Ponten et al., 2007). With the reliance of seizure spread on network topology, epileptogenesis can occur as a result of disruptions to functional and structural connectivity (Avanzini and Franceschetti, 2009; Terry et al., 2012; Fornito et al., 2015). Functional connectivity mapping in stroke patients often reveals hyperconnectivity, where functional network components paradoxically show an increase in connectivity post-stroke (Hillary and Grafman, 2017). Up to 10% of stroke patients suffer seizures either early or late in their recovery and currently there exists no reliable prognostic tool for determining if these will develop into epilepsy (Myint et al., 2006). Unfortunately, application of AEDs as a preventative measure has thus far proven ineffective, indicating that epileptogenesis is partially independent of neuronal excitability or subject to interference from complex homeostatic mechanisms (Gilad et al., 2011). These forms of acquired epilepsy highlight the heterogeneity of the disorder and the necessity of a combined network and dynamics analysis. The application of criticality analysis to epilepsy disorders can potentially act as both a trial and guideline for other neurological disorders, such as psychiatric (van Bokhoven et al., 2018), developmental (Tinker and Velazquez, 2014; Gao and Penzes, 2015; Li L. et al., 2018), and degenerative disorders (Jiang et al., 2018; Ren et al., 2018; Marcuzzo et al., 2019).

Still, caution must be taken when comparing these studies of macroscale dynamics to their underlying meso and microscale mechanisms (Meisel and Kuehn, 2012). Network scale is a crucial feature of these mechanisms, and node and edge descriptors at different scales substantially alter the relevant activity dynamics. Additionally, the highly divergent methods for avalanche detection between different assessment modalities [macroscale: fMRI, (i)EEG, MEG, ECoG; mesoscale: spikes and LFPs from tetrodes and MEAs] risk erroneous conflation of results between different scales. Each method of avalanche detection and definition requires a thorough investigation into its relevance and robustness to multiple tests. In the final section of this paper we will highlight steps to improve the accuracy and standardization of criticality analysis, as well as the relationship between structural and critical dynamics.

## Conclusion

Network neuroscience has seen explosive growth in the clinical field within the past two decades, providing insight into pathophysiology and disease propagation. However, such rapid growth comes along with the challenge of standardizing the measures applied (Hallquist and Hillary, 2018). The current lack of standardized graph theory measures has created a widely dissimilar range of network definitions and graph metrics to the point where it precludes meta-analysis. Advancements in the empirical study of criticality in neural systems are also beginning to see rapid growth, and it can be a struggle to keep up with which measures are best to apply. The consolidation and standardization of metrics used in the study of critical dynamics and connectivity in neural networks thus remains a considerable challenge, yet the sooner this challenge is approached, the more it can be mitigated. Therefore, we have highlighted certain measures that have proven useful in the study of criticality in the context of neuroscience (see Box 2). Given the complex and multifaceted nature of criticality, we fully expect later studies will further expand upon and improve measures. However, applying and comparing the same measures across experiments will lessen the future burden of comparison.

Criticality holds the promise of bridging several scales of neural activity by its nature as a scale-free property. Yet as we have examined, the step from statistical models of criticality to experimental analysis is a difficult one, where constraints and complicating factors arising from experimental methods and the underlying biological mechanisms make themselves known. As we have discussed, the different recording modalities across scales apply disparate approaches to analyzing criticality, making any comparison fraught with analytical pitfalls, and this issue may be further exacerbated by more indirect measures such as fMRI with its different timescales. While there are some groups (Gireesh and Plenz, 2008; Miller et al., 2019) making substantial progress on these experimental issues, as more and more groups turn toward applying criticality-based measures in the clinic and laboratory they need to be cognizant of the intricacies inherent in these topics. Similarly, intuitions from theoretical work, the idea that epileptic systems are supercritical and thus should have branching ratios exceeding 1, can be counter to experimental findings (Hobbs et al., 2010; Plenz, 2012), necessitating a closer look at analytical techniques and theoretical understandings.

Throughout this review, we have attempted to highlight the multifaceted nature of criticality and the potential its analysis holds as a metric of network health. Criticality is closely tied to the efficiency of its underlying network structure, as this structure supports the propagation of dynamical activity through the system. The emergence of these efficient topologies in turn results from the dynamics of the structure itself: the changes in connectivity mediated by the local and global plasticity. This intertwining of criticality and structural dynamics is an essential feature of the critical state, and examining in isolation any single feature contributing to the behavior of a network may forgo the complex interplay that gives rise to critical dynamics. Neural networks organize into small-world and hierarchical modular topologies in part to support critical dynamics, and both structure and dynamics likely develop due to the computational benefits they afford. Furthermore, there is evidence that networks in the critical state display characteristics indicative of their optimal computational capacity, yet few studies have explicitly focused on highlighting these benefits conferred by criticality. A focus on this aspect of criticality would also aid in understanding what goes wrong—or what computational functions may be affected—when a network is damaged or diseased. In the future, we hope more studies take into consideration the interplay between structure and critical dynamics, as well as the functional benefits this confers, as criticality analysis and network neuroscience can provide significant insight into complexity, computation, and medicine.

## Author Contributions

KH and OH developed the focus and organization of the manuscript. KH and VF prepared the figures. All authors contributed to the choice of review topic and the writing and review of the manuscript.

## Conflict of Interest

The authors declare that the research was conducted in the absence of any commercial or financial relationships that could be construed as a potential conflict of interest.
